# Circadian Rhythm and Cartilage Extracellular Matrix Genes in Osseointegration: A Genome-Wide Screening of Implant Failure by Vitamin D Deficiency

**DOI:** 10.1371/journal.pone.0015848

**Published:** 2011-01-11

**Authors:** Cristiane Machado Mengatto, Federico Mussano, Yoshitomo Honda, Christopher S. Colwell, Ichiro Nishimura

**Affiliations:** 1 Division of Advanced Prosthodontics, Biomaterials and Hospital Dentistry, The Jane and Jerry Weintraub Center for Reconstructive Biotechnology, University of California Los Angeles School of Dentistry, Los Angeles, California, United States of America; 2 Department of Prosthodontics and Periodontology, Piracicaba School of Dentistry, State University of Campinas, Piracicaba, Sao Paulo, Brazil; 3 Department of Biomedical Science and Human Oncology, University of Turin, Turin, Italy; 4 Department of Psychiatry and Biobehavioral Science, David Geffen School of Medicine at University of California Los Angeles, Los Angeles, California, United States of America; University of Bergen, Norway

## Abstract

**Background:**

Successful dental and orthopedic implants require the establishment of an intimate association with bone tissue; however, the mechanistic explanation of how biological systems accomplish osseointegration is still incomplete. We sought to identify critical gene networks involved in osseointegration by exploring the implant failure model under vitamin D deficiency.

**Methodology:**

Adult male Sprague-Dawley rats were exposed to control or vitamin D-deficient diet prior to the osteotomy surgery in the femur bone and the placement of T-shaped Ti4Al6V implant. Two weeks after the osteotomy and implant placement, tissue formed at the osteotomy site or in the hollow chamber of T-shaped implant was harvested and total RNA was evaluated by whole genome microarray analyses.

**Principal Findings:**

Two-way ANOVA of microarray data identified 103 genes that were significantly (>2 fold) modulated by the implant placement and vitamin D deficiency. Kyoto Encyclopedia of Genes and Genomes (KEGG) analyses assigned the highest *z*-score to the circadian rhythm pathway including neuronal PAS domain 2 (NPAS2), and period homolog 2 (Per2). NPAS2 and Aryl hydrocarbon receptor nuclear translocator-like (ARNTL/Bmal 1) were upregulated around implant and diminished by vitamin D deficiency, whereas the expression pattern of Per2 was complementary. Hierarchical cluster analysis further revealed that NPAS2 was in a group predominantly composed of cartilage extracellular matrix (ECM) genes. Whereas the expression of bone ECM genes around implant was not significantly affected by vitamin D deficiency, cartilage ECM genes were modulated by the presence of the implant and vitamin D status. In a proof-of-concept *in vitro* study, the expression of cartilage type II and X collagens was found upregulated when mouse mesenchymal stem cells were cultured on implant disk with 1,25D supplementation.

**Conclusions:**

This study suggests that the circadian rhythm system and cartilage extracellular matrix may be involved in the establishment of osseointegration under vitamin D regulation.

## Introduction

Increasing numbers of dental and orthopedic procedures require the implantation of foreign materials into bone to replace missing or damaged body parts. These implants are made of mechanically sustainable metals such as titanium (Ti) that may be treated to generate surface microtopography or coated with calcium phosphates such as hydroxyapatite (HA). To be successful and functional, these endosseous implants have to establish an intimate integration of bone and implant that is referred to as osseointegration [1], [2]. To date, the mechanisms of osseointegration have been largely explored from the prospective of bone wound healing [3], [4], [5]. However, recent investigations have indicated that the placement of implant into bone activates a wide range of biological reactions [6], [7], [8], [9], not limited to those of bone remodeling. Therefore, osseointegration may be a complex process involving a number of mechanistic systems, some of which have not yet been identified.

While the prevalence of implant failure is small in dentistry [10], [11], understanding the biological basis for these rare cases should provide novel insights into the mechanisms underlying osseointegration. For example, a striking rate of implant failure has been reported in X-linked hypophosphatemic rickets (XLH) patients [12]. XLH is an X-linked dominant disorder highlighted by renal defects in phosphate reabsorption with various bone manifestations (Online Mendelian Inheritance in Men, #307800). XLH is associated with an incomplete defect in the regulation of 25-hydroxyvitamin D-1-alpha-hydroxylase, a critical enzyme to activate vitamin D [13]. Separately, Kelly et al. (2009) reported that the rate of implant osseointegration characterized by the upholding mechanical load and histological bone to implant contact ratio was significantly impaired in vitamin D-deficient rats [14]. Vitamin D is a steroid hormone that participates in broad physiological actions [15], [16], [17], [18]. Abnormal vitamin D-related regulations have resulted in skeletal diseases such as rickets and osteomalacia [19], [20], catabolic bone remodeling [20], [21], [22], autoimmune diseases [23], and increased susceptibility to infectious diseases [24]. However, the role of vitamin D in the establishment of osseointegration has not been addressed.

Based on these previous data, we have hypothesized that the critical biological mechanism responsible for osseointegration may, in part, be dependent upon vitamin D. The exploration of how vitamin D deficiency alters osseointegration should provide a unique experimental platform to elucidate its mechanisms. Furthermore, this work has a clinical relevance as the prevalence of vitamin D deficiency has been reported increasing in the populations worldwide [25], [26], [27]. Recently, the prevalence of vitamin D deficiency was found to be high even in the adolescent population with disproportionate burden falling on the non-Hispanic blacks [28]. The present study was designed to broadly characterize the transcriptional changes that occur as a result of implant placement into the bone of vitamin D-deficient and control rats using a whole genome microarray screening. We report here an unexpected identification of a gene network composed of circadian rhythm systems and cartilage extracellular matrix genes, which may participate in the establishment of osseointegration under vitamin D regulation.

## Results

### Impaired osseointegration in vitamin D deficient rats

**Figure 1:**
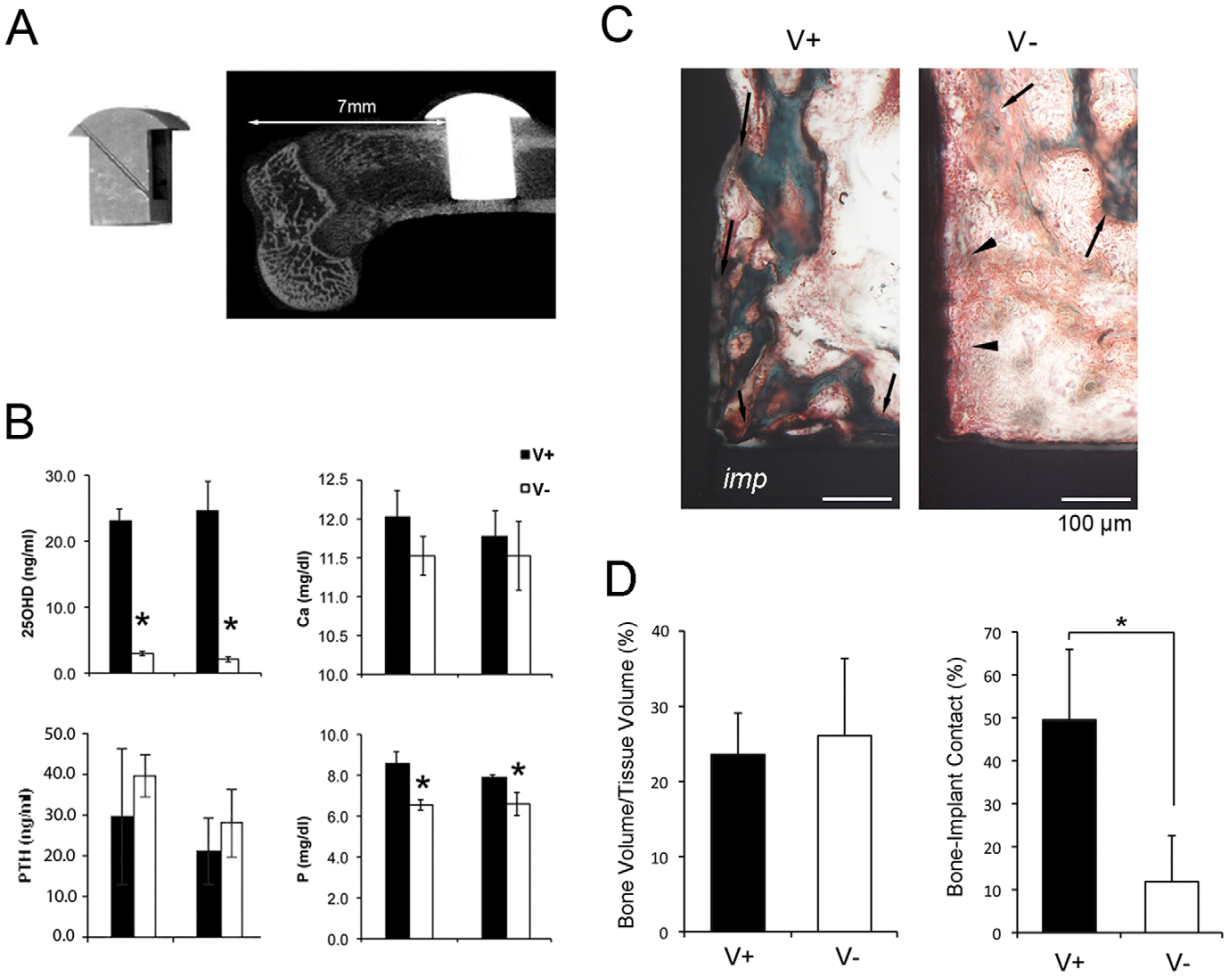
T-shaped implant placed in vitamin D sufficient (V+) and deficient (V-) rats. **A**. T-shaped experimental implant with a hollow inner chamber was fabricated with Ti6Al4V and the implant surface was treated with dual acid-etching and discrete deposition of HA nanoparticles. T-shaped implant was placed in the osteotomy site of the distal end of rat femur. **B**. Serum chemistry evaluations of 25-hydroxy vitamin D3 (25D), parathyroid hormone (PTH), calcium (Ca), and phosphorous (P). *: p<0.05. **C**. Non-decalcified histology of bone tissues grew in the inner chamber of T-shaped implant. The bone tissue (arrows; green in Goldner's Masson Trichrome staining) was closely adhered to the implant surface in V+ rats. In V- rats, the intimate bone-implant was not observed; and instead, implant surface was associated with fibrous connective tissue (arrowheads; red staining). **D**. Histomorphometric characterization of bone volume and bone-implant contact within the inner chamber. *p<0.05.

The present study used Sprague-Dawley rats that were subjected to the vitamin D deficient protocol [29] prior to the implant placement and throughout the study. Both vitamin D sufficient control (V+) and deficient (V-) rats received T-shaped experimental implant with a hollow inner chamber (**Figure 1A**) in the femur (ITV+ and ITV- groups). Blood serum analyses demonstrated the significant reduction of circulating precursor vitamin D (25-hydroxy vitamin D3: 25D) with hypophosphoremia in the vitamin D deficient (V-) group. There was a tendency of increased serum parathyroid hormone and decreased serum calcium level in the V- group (**Figure 1B**), whereas serum levels of magnesium, triglycerides and glucose were not affected (data not shown).

T-shaped implant has been used for the stringent evaluation of implant-induced *de novo* tissue formation and osseointegration [30], [31]. The implant surface was treated with dual acid-etching and HA nanoparticles [32], which has been shown more sensitive to vitamin D-dependent regulation [14] and thus should be suitable for the present study. Non-decalcified histology of ITV+ rats indicated the establishment of osseointegration in the implant hollow chamber (**Figure 1C**). The morphometric measurement of bone volume as well as bone-implant contact (**Figure 1D**) was equivalent to recently reported data with the similar implant surface characteristics [30]. Strikingly, while the volume of bone growth in the implant hollow chamber of the ITV- group was similar to that of the control ITV+ group, the bone to implant contact was significantly impaired in the ITV- group (**Figure 1D**). These observations suggest that the implant and bone integration critical for the establishment of osseointegration was significantly impaired by vitamin D deficiency in this model.

### Two-way ANOVA analysis

Two-way ANOVA with Benjamini and Hochberg correction revealed 103 transcripts that were differently expressed under the studied conditions. The affected genes were differentially expressed under healing effect of with or without implant. This trend was generally consistent with the previous study [9] and thus validated our model. The two-way ANOVA analysis further identified 4 transcripts whose expression was altered by vitamin D deficiency (*P*<0.05) and 3 transcripts whose expression was altered by the interaction between vitamin D deficiency and implant factors (*P*<0.05) (**Table 1**). Among the VD-deficiency altered-gene list, we found Neuronal PAS domain 2 (NPAS2) and Period Homolog 2 (Per2) as the most significantly affected genes by vitamin D deficiency in the peri-implant tissue (**Table 1**).

**Table 1:** Significantly modulated genes identified by Two-way ANOVA for the vitamin D deficiency effect.

Effects	Gene Title	Gene ID
**Vitamin D**	Neuronal PAS domain 2 (NPAS2)	CO386194
	Period homolog 2 (Per2)	NM031678
	Visinin-like 1	NM_012686
	TSC22d3 (GILZ)	AI029054, NM031345
**Implant-Vitamin D Interaction**	CEA-related cell adhesion molecule 1	BF420163
	CD226 antigen	XM_225664
	Copine IV, transcript variant 3	XM_001070003

### KEGG analysis

The possible relation, co-regulation and function of 103 altered genes were further investigated by the Kyoto Encyclopedia of Genes and Genomes (KEGG) analyses. KEGG Pathway is a collection of online databases for systematic analysis of gene functions in the known molecular pathways [33]. The KEGG analysis revealed genes involved in 6 distinct pathways for the genes differently expressed under healing effect, and 1 pathway that were over-represented when we considered the effect of V- alone (**Table 2**). These identified KEGG pathways included: circadian rhythm, formation of extracellular matrix (ECM-receptor interaction, arginine and proline metabolism, and glycerolipid metabolism), and xenobiotics biodegradation and metabolism (1- and 2-Methylnaphthalene degradation) (**Table 2**). Among these pathways, it was found that circadian rhythm was most significantly altered by implant placement (z-score: 7.65) and V- condition (*z*-score: 22.81).

**Table 2:** Representative KEGG Pathways with z-score >2.0.

Effects	KEGG Pathway	z-score
**Implant**	Circadian rhythm	7.65
	Neuroactive ligand-receptor interaction	4.64
	1- and 2-Methylnaphthalene degradation	3.34
	Arginine and proline metabolism	2.99
	ECM-receptor interaction	2.65
	Glycerolipid metabolism	2.50
**Vitamin D**	Circadian rhythm	22.81

### Hierarchical Cluster analysis revealed a gene network containing NPAS2 and cartilage matrix genes

**Figure 2:**
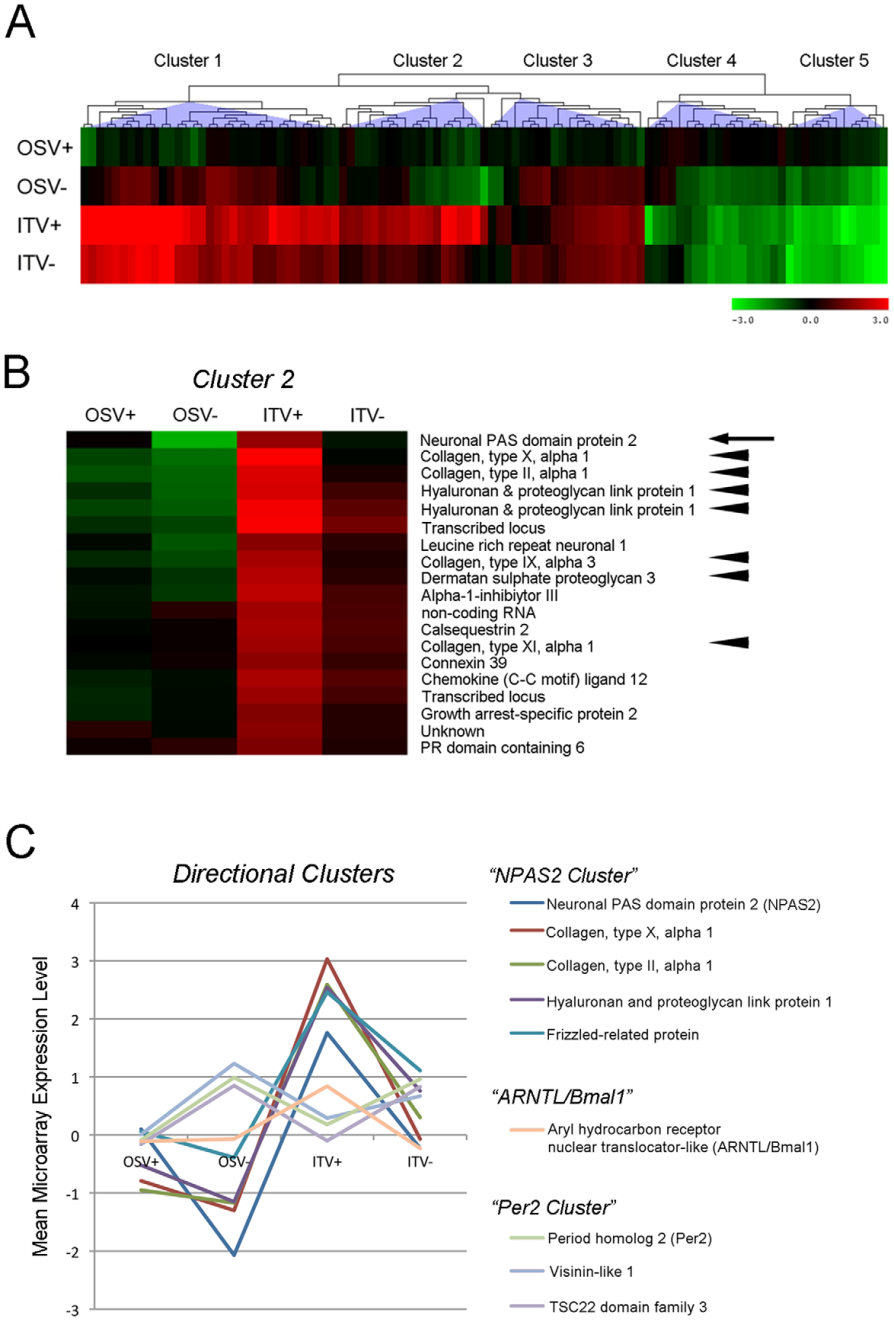
Hierarchical cluster analysis. **A**. Two-way ANOVA resulted in a total of 103 genes, which were further grouped into 5 clusters (Cluster 1 to 5). **B**. Cluster 2 contained NPAS2 (arrow), one of the most significantly affected genes, as well as cartilage ECM genes (arrowheads). **C**. Directional hierarchical clustering revealed that the NPAS2 and Per2 clusters behaved in a complementary fashion.

The transcripts that were statistically different and had above 2.0-fold change in the two-way ANOVA analysis were grouped into hierarchical clusters (**Figure 2A**). The analysis resulted in 5 hierarchical clusters with various sizes. Each cluster contained at least 1 gene of transcripts that were identified by the KEGG analysis. The clusters 1 to 3 were composed of genes that were upregulated by the implant placement, whereas the clusters 4 and 5 contained downregulated genes by the implant placement. In general, vitamin D deficiency attenuated the implant-induced gene modulation.

The cluster 2 contained the transcripts most significantly influenced by vitamin D deficiency, which consisted of 19 genes including NPAS2 and the disproportionately large representation of cartilage extracellular matrix (ECM) genes such as type II, type X and type IX collagen alpha chains, hyaluronan-proteoglycan link protein and dermatan sulfate proteoglycan 3 (**Figure 2B**). The cluster 3 contained Per2 and Aryl hydrocarbon receptor nuclear translocator-like (ARNTL, a.k.a Bmal1).

The hierarchical clustering re-evaluated by directionality indicated that upregulated NPAS2 and cartilage ECM genes by the implant placement were completely attenuated by vitamin D deficiency. On the contrary, the cluster containing Per2 was upregulated by vitamin D deficiency and showed a complementary expression pattern to the NPAS2 cluster (**Figure 2C**).

### Expression of cartilage and bone extracellular matrix-related genes

**Figure 3:**
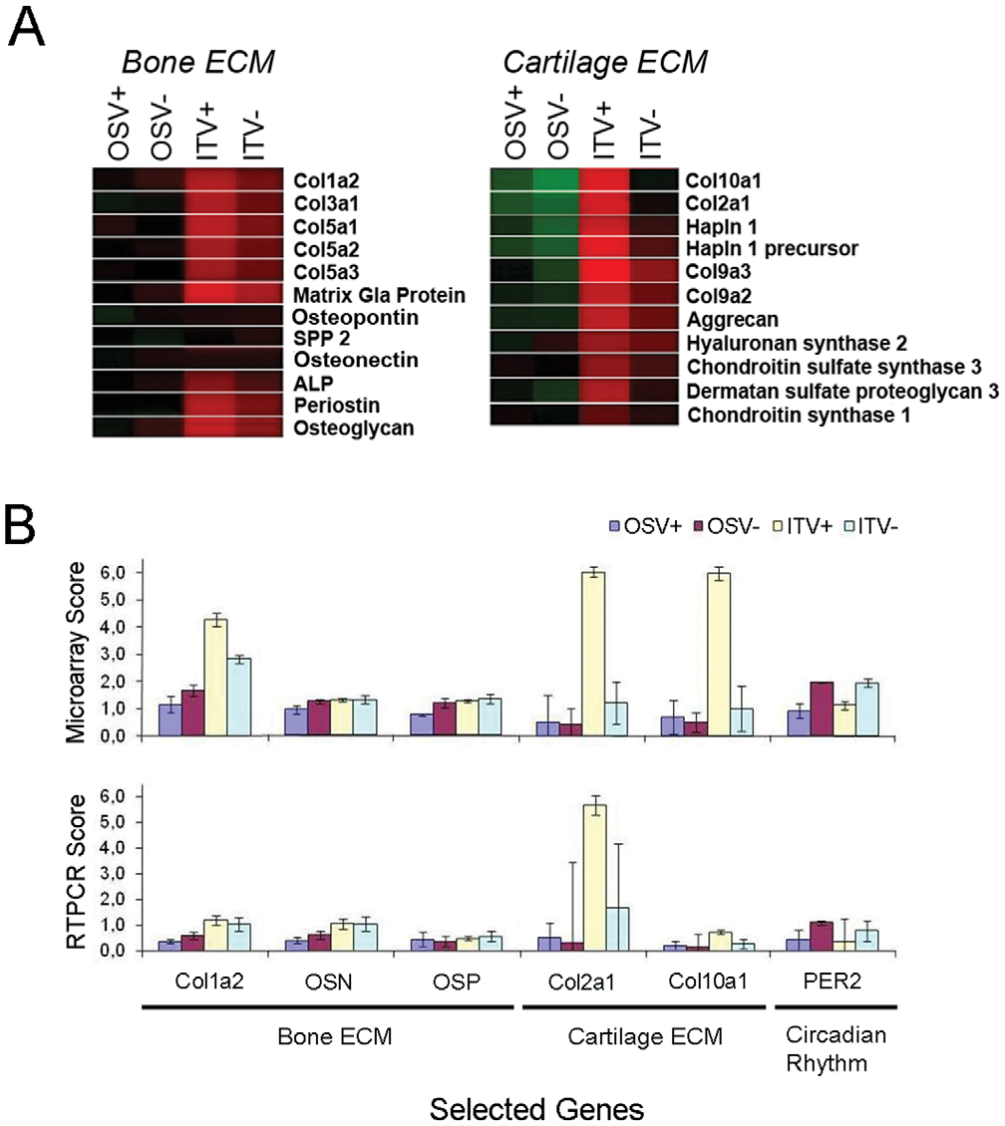
Expression of bone and cartilage extracellular matrix (ECM). **A**. Compiled microarray data of bone and cartilage ECM genes. Cartilage ECM genes were more significantly affected by implant placement and vitamin D deficiency than bone ECM genes. **B**. Microarray data for bone and cartilage ECM genes were confirmed by real time reverse transcription polymerase chain reaction (RTPCT).

The hierarchical cluster analysis revealed the potential close association between NPAS2 and cartilage ECM genes. When the microarray data of ITV+ and ITV- groups were re-evaluated by t-test without Benjamini and Hochberg correction, it was found that nearly all cartilage ECM-related genes were significantly down-regulated in the ITV- group, whereas none of bone ECM-related genes were affected (**Figure 3A** and **Table 3**). The microarray data were confirmed by RTPCR (**Figure 3B**).

**Table 3:** Unadjusted t-test of significantly modulated genes between ITV+ (n = 4) and ITV- (n = 4).

Gene Groups	Gene Name	Ratio*	P-value
**Circadian Rhythm-Related**	Neuronal PAS domain 2 (NPAS2)	3.96 D	0.00028
	Period homolog 2 (Per2)	1.73 U	0.00852
	Aryl hydrocarbon receptor nuclear translocator-like (ARNTL)	2.10 D	0.00071
	Adrenergic receptor, alpha 2c	2.12 D	0.03321
**Cartilage ECM-Related**	Type II collagen alpha 1	4.88 D	0.02847
	Type IX collagen alpha 1	n.d.**	--
	Type IX collagen alpha 2	2.00 D	0.02495
	Type IX collagen alpha 3	4.65 D	0.01270
	Type X collagen alpha 1	12.47 D	0.01994
	Type XI collagen alpha 1	2.04 D	0.04079
	Hyaluronan and proteoglycan link protein 1 (Hapln1)	3.43 D	0.03612
	Dermatan sulfate proteoglycan 3	3.15 D	0.00980
	Aggrecan	2.07 D	0.01696
	Sox9	n.d.	--
	Catenin	n.d.	--
**Bone ECM-Related**	Type I collagen alpha 1	n.d.	--
	Type I collagen alpha 2	n.d.	--
	Type V collagen alpha 1	n.d.	--
	Type V collagen alpha 2	n.d.	--
	Type V collagen alpha 3	n.d.	--
	SPP1 (Osteopontin)	n.d.	--
	Osteocalcin	n.d.	--
	SPARC (Osteonectin)	n.d.	--
	Matrix Gla Protein	n.d.	--
	Alkaline phosphatase	n.d.	--
	Runx2	n.d.	--
**Hypoxia-Related**	Hypoxia-inducible factor 1 alpha	n.d.	--
	Heat shock protein 1	1.61 D	0.03421

*Average gene expression ratio from microarray data analyzed by GeneSifter. D =  down-regulated in ITV-; U =  up-regulated in ITV-.

**n.d.  =  not detected within the threshold of 1.5-fold change.

### Increased synthesis of cartilage matrix molecules by mouse bone marrow mesenchymal stem cells cultured with implant disk and vitamin D supplementation *in vitro*

**Figure 4:**
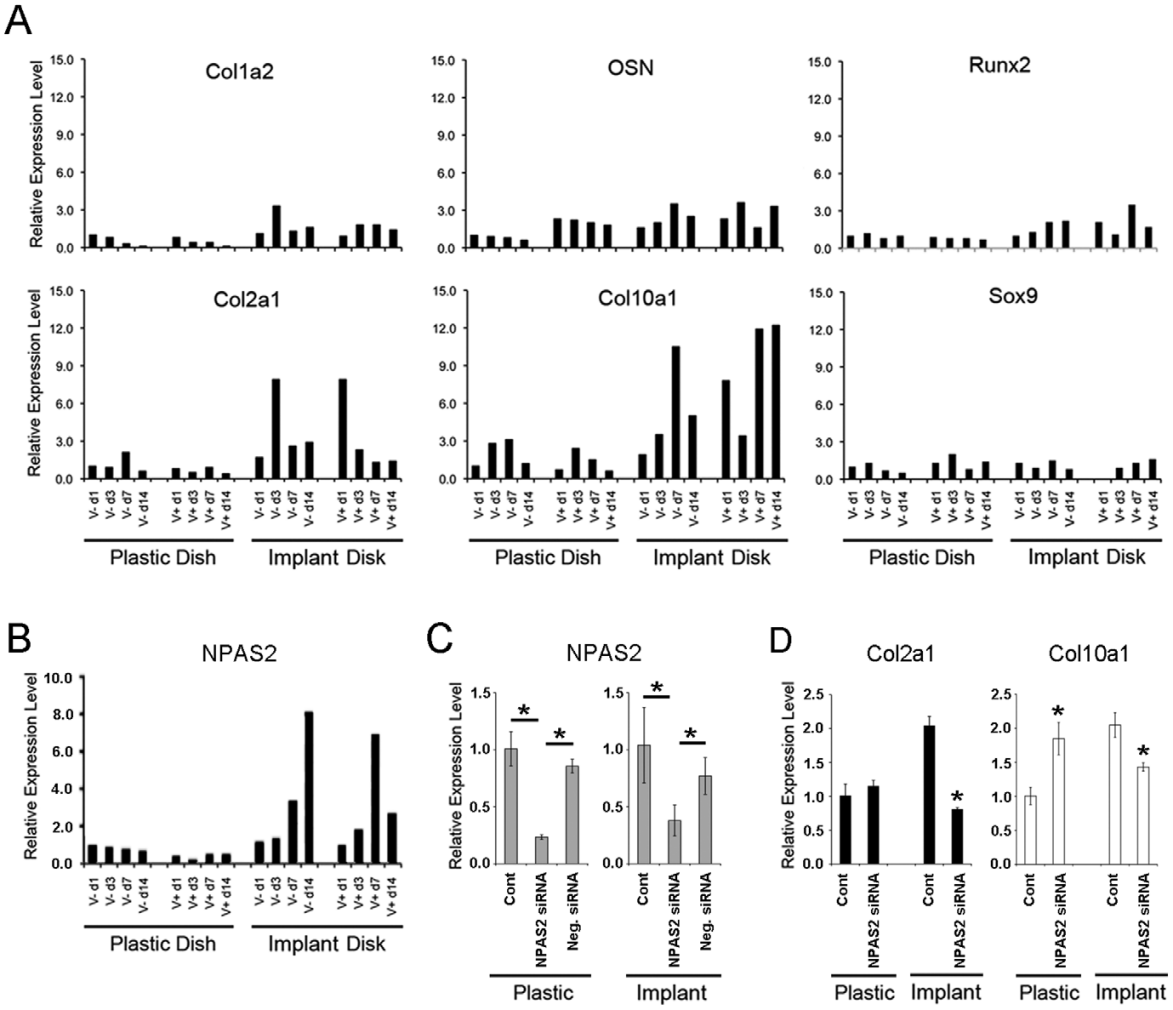
The effect of implant disk and 1,25D supplementation on mouse bone marrow stromal cells (D1 cells) *in vitro*. **A**. D1 cells cultured on polystyrene dish or implant disk with or without 1,25D supplementation (V+ and V−, respectively) were subjected to RT-PCR evaluation of bone-and cartilage-related genes. **B**. The steady state mRNA levels of NPAS2 were assessed by RT-PCR under the same conditions. **C**. The commercially available siRNA effectively knocked down the targeted NPAS2 in D1 cells cultured on polystyrene dish or implant disk with 1,25D supplementation. The RT-PCR data were evaluated against the average control value in each group. *: p<0.05. **D**. The effect of NPAS2 knockdown on the expression of type II and type X collagens. The RT-PCR data were evaluated using the average control value of D1 cells cultured on plastic dish as the reference. *: p<0.05, against the control in each group.

To investigate the observed upregulation of cartilage ECM genes in the peri-implant tissue, a proof-of-concept *in vitro* study was performed. The expression of cartilage ECM-related genes such as type II and type X collagens was significantly upregulated when mouse bone marrow mesenchymal stem cells (D1 ORL UVA [D1]; D1 cells) were cultured with implant disk (**Figure 4A**). Furthermore, the 1,25-dihydroxyvitamin D (1,25D) supplementation accelerated the timing of type II and type X collagen expression, whereas the expression of Sox9 was not affected by implant disk nor 1,25D supplementation (**Figure 4A**). In contrast, the expression of bone ECM-related genes such as type I collagen, osteonectin and Runx2 (**Figure 4A**) as well as osteocalcin and osteopontin (data not shown) was only modestly increased with the presence of implant disk. The 1,25D supplementation also showed mild effect on the expression of bone matrix-related genes.

The presence of implant disk and 1,25D supplementation increased the expression of NPAS2 *in vitro*; however, their effect was not clearly observed during the early culture periods (**Figure 4B**). We examined if there was any association between NPAS2 and the increased expression of cartilage ECM; i.e., type II and type X collagens. The application of a commercially available siRNA targeting NPAS2, not the negative control siRNA, demonstrated approximately 70∼80% reduction of the steady state NPAS2 mRNA level (**Figure 4C**). The siRNA-derived NPAS2 knockdown resulted in the significant decrease of the type II and type X collagen mRNA levels, when D1 cells were maintained in the 1.25D supplemented culture medium with implant (**Figure 4D**). In fact, NPAS2 knockdown normalized the elevated type II collagen expression induced by the presence of implant disc. On the contrary, the NPAS2 siRNA treatment did not completely normalize the type X collagen expression of D1 cells cultured with implant disc. D1 cells cultured without implant, NPAS2 knockdown did not affect type II collagen expression; but unexpectedly, increased the type X collagen mRNA level.

**Figure 5:**
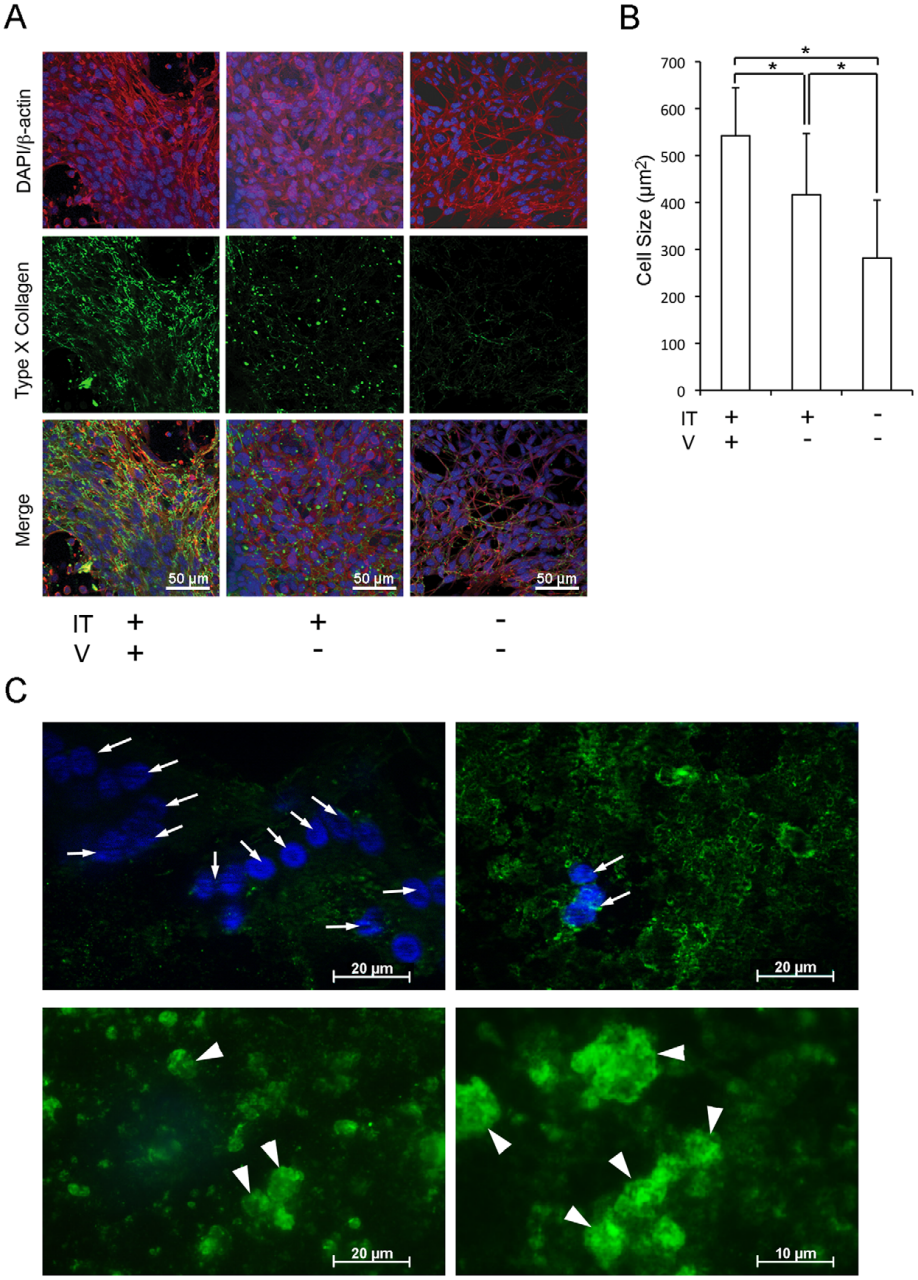
Immunological identification of type X collagen associated with implant osseointegration. **A**. Mouse bone marrow stromal cells (D1 cells) were cultured on implant disk (IT) or glass dish with or without 1,25D supplementation (V). Confocal laser scanning micrographs depicted cell nuclei (DAPI: blue), cytoskeleton (β-actin: red) and type X collagen (green). **B**. The size of D1 cells was determined by the β-actin positive area per nucleus. The average cell size under different culture conditions was compared. *: p<0.05. **C**. T-shape implants were harvested 2 weeks after the placement in femur of V+ rats and bone tissues were carefully removed from the external implant surface. In the highly cellular region depicted by a cluster of nuclei (arrows; DAPI), type X collagen was not observed (upper left panel). In the transition region, type X collagen (green) showed a similar appearance as in the *in vitro* culture (upper right panel). In the areas of exposed bone-implant interface, where no cells remained (lower panels), type X collagen appeared to be involved in extracellular matrix with defined hexagonal structures (arrowhead).

Immuno-identification of type X collagen network on the implant disk was positively associated with 1,25D supplementation (**Figure 5A**). While identifiable, type X collagen network was less developed without 1,25D supplementation; however, when D1 cells were cultured without the implant disk, type X collagen was not detected. The average cell size was increased by the placement of implant disk and 1,25D supplementation (**Figure 5B**).

In an additional experiment, we further evaluated the presence of type X collagen in the interacting tissues with implant. T-shape implants were harvested from the femur of control rats 2 weeks after the surgical placement and the external implant surface was immunohistologically evaluated after major bone tissues were removed. Confocal laser scanning microscopy clearly detected the presence of type X collagen epitopes and found that these epitopes were disproportionately localized in the presumptive intermediate tissue between bone and implant (**Figure 5C**). Where clusters of cells still remained, type X collagen was either absent or showed a positive staining similar in appearance to that found in the *in vitro* study. In the area of presumptive bone-implant interface, type X collagen appeared to be organized into the well-defined extracellular matrix with a hexagonal structure (**Figure 5C**).

## Discussion

This study, for the first time, provided the stringently determined network of genes that were associated with success and failure of implant osseointegration. The novel design of the present investigation was to comparatively evaluate the whole genome microarray associated with implant placement in vitamin D-deficient and control rats. Initially, we anticipated finding genes that were involved in the classic category of bone remodeling; however, in two-way ANOVA, we found NPAS2 as the most significantly affected gene by vitamin D deficiency in the peri-implant tissue. NPAS2 is a member of the basic helix-loop-helix-PAS family of transcription factors, involved in the regulation of circadian rhythm [34]. The primary molecular target of NPAS2 is Per2, which was also found to be significantly modulated by vitamin D deficiency in the peri-implant tissue (**Table 1**).

Furthermore, KEGG (**Table 2**) pathway analyses also highlighted the potential involvement of the circadian rhythm system in the establishment of osseointegration. Circadian oscillations are generated by a core set of genes: NPAS2, Per2 as well as ARNTL/Bmal1 that are part of a negative feedback loop and are referred to as “clock” genes [34]. These genes are widely expressed throughout the body tissues and are likely involved the temporal patterning of local gene transcription [35], [36]. In the present study, NPAS2 was upregulated in the peri-implant tissue, which was completely attenuated under the vitamin D-deficient condition. In contrast, the vitamin D-deficient environment significantly increased the expression of Per2 (**Table 3**). The reevaluation of microarray data of ITV+ and ITV- groups by *t*-test further identified Arntl/Bmal1 that was significantly downregulated in the ITV- group (**Table 3**).

Previous work has shown the rhythmic expression of clock genes in osteoblasts and found that mutant mice lacking these clock genes exhibit altered osteoblast proliferation [37]. Other studies have found evidence for circadian regulation of cell cycle and cell proliferation [38], [39]. In the present study, we found the evidence that the placement of the implant into bone alters the expression of genes involved in circadian regulation. The experimental implant was fabricated from Ti alloy with submicron surface topography. Approximately 50% of the implant surface was further modified by HA nanoparticles [30], [32], [40], [41]. Ti implants have been long considered bio-inert; however, recently, Ogawa and colleagues have demonstrated that UV treatment of Ti implant can activate the surface energy, which underscores the functional involvement of electrostatic mechanism [42]. The implant surface further contains complex carbon molecules such as polyaromatic hydrocarbons (PAHs) [43]. It may be possible that the implant material could induce biological responses such as electrostatic or xenobiotics responses. NPAS2, Per2 and Arntl/Bmal1 responsible for circadian rhythm control contain PAS (Per-Arnt-Sim) motifs. It has been suggested that PAS-containing transcription factors are also responsible for the adaptation to environmental cues such as the metabolism of xenobiotics and hypoxia signaling [44], [45]. It is tempting to speculate that the microenvironment induced by the placement of implant may influence the disproportionate up-regulation of circadian rhythm genes, in particular NPAS2.

More surprising observation was the extent to which vitamin D deficiency also profoundly altered the expression of circadian rhythm-related genes. Previous work has found evidence that transcript for a vitamin D receptor is expressed with a daily rhythm, at least in brown adipose tissue [46]. Still, the pervasive impact of implant placement and vitamin D deficiency on circadian rhythm-related gene expression in the bone matrix is a novel finding.

Hierarchical clusterization is a holistic approach based on the calculation and comparison of pairwise correlation coefficients between two datasets to arrange genes according to similarity or closeness in expression pattern [47]. NPAS2 and seemingly unrelated cartilage ECM genes such as col2a1, aggrecan, and col10a1 were co-localized in a small cluster (**Figure 2B**). This finding was unexpected, but could suggest a novel co-regulatory mechanism affecting skeletal circadian rhythm system and cartilage ECM gene expression. Bone wound healing associated with implant osseointegration undergoes intramembranous bone formation without cartilage precursor tissue [3], [4]; therefore the expression of cartilage ECM molecules during osseointegration was a puzzling observation. RNA samples used for microarray assay were reevaluated by RTPCR and confirmed the expression of cartilage matrix ECM genes (**Figure 3B**); however, histological cartilage tissue was not formed in the inner chamber of T-shaped implant (**Figure 1C**).

Under hypoxic *in vitro* conditions, the expression of cartilage ECM genes was reported to increase in bone marrow mesenchymal stem cells, which expressed Sox9, an initiation factor of chondrogenic differentiation [48], [49]. Hypoxia-induced factor 1 alpha (HIF-1a) has been shown to mediate the hypoxia-induced chondrogenic differentiation [50], [51]. Thus, we initially postulated that implant placement might induce a relatively hypoxic local microenvironment, inducing the observed upregulation of cartilage ECM genes. However, reevaluation of the microarray data revealed that the expression levels of HIF-1a and Sox9 were not significantly affected in all groups (**Table 3**). Thus, the upregulation of cartilage ECM genes associated with implant healing may employ different mechanisms.

The expression of cartilage ECM during the establishment of osseointegration has not been reported. We designed and performed a proof-of-concept *in vitro* experiment to determine if the placement of the implant affects the expression of cartilage ECM. Mouse bone marrow D1 cells, when cultured on implant disk with 1,25D supplementation, significantly accelerated the expression of type II and type X collagens (**Figure 4A**). Although the expression pattern of NPAS2 in this *in vitro* system did not match with those of type II and type X collagens (**Figure 4B**), the siRNA-derived knockdown study indicated that NPAS2 appeared to regulate their expression (**Figure 4D**). Circadian rhythm molecule ARNTL/Bmal1 is the obligatory partner of NPAS2 to induce target genes through a cis-acting MOP3/MOP4 responsive element (M34RE; CACGTGACC) [44]. It has been reported that type II and type X collagen genes as well as aggrecan gene contain active M34RE [52], [53]. As such, we postulate that the ectopic synthesis of cartilage ECM molecules around the implant may be regulated by the activated NPAS2-ARNTL/Bmal1 heterodimer. However, an unexpected discrepancy was found in the type X collagen expression modulated by NPAS2 knockdown when D1 cells were cultured without implant. We speculate that NPAS2-ARNTL/BmalI may regulate this gene as either an enhancer or a silencer and the implant placement may disproportionately activate its anabolic function.

The presence of type X collagen protein associated with implant substrate *in vitro* (**Figure 5A**) and *in vivo* (**Figure 5C**) further provides the supporting evidence that the presence of implant material may alter the cellular behavior and induce the ectopic synthesis of cartilage ECM molecules. With 1,25D supplementation, D1 cells on implant disk further spread to form more polygonal than spindle shapes. Moreover, the type X collagen found in the presumptive bone-implant interface showed a well-defined hexagonal structure (**Figure 5C**). Kwan et al. (1991) reported that type X collagen assembled into a mat-like structure *in vitro*, and the prolonged incubation resulted in the formation of a hexagonal lattice [54]. Therefore, although macromolecular organization of type X collagen has not been reported *in vivo*, the ECM structure found in the presumptive bone-implant interface may indeed contain type X collagen. We speculate that the postulated ectopic synthesis of cartilage ECM may play a previously unreported role in the establishment of osseointegration.

### Conclusions

The present genome-wide microarray study of implant ossoeintegation suggests that the specific microenvironment induced by implant placement significantly affect a spectrum of gene expression networks, possibly including peripheral circadian rhythm mechanisms. The potential interaction between NPAS2 and cartilage matrix genes has led to a hypothetical mechanistic model of osseointegration that through the circadian rhythm-related system, bone marrow mesenchymal cells may initiate ectopic synthesis of cartilage matrix molecules including type X collagen without the formation of *in situ* cartilage tissue. Vitamin D deficiency negatively affects these processes, resulting in the lack of bone and implant integration. Taken together, we postulate that the establishment of osseointegration requires the peripheral circadian rhythm system, which transiently activates the synthesis of a selected set of cartilage matrix molecules possibly guiding the intimate integration of implant surface and bone tissue.

## Materials and Methods

This study was carried out in strict accordance with the recommendations in the Guide for the Care and Use of Laboratory Animals of the National Institutes of Health. The protocol was approved by the University of California at Los Angeles Chancellor's Animal Research Committee (ARC 1997-136). All surgery was performed under isoflurane inhalational anesthesia, and all efforts were made to minimize suffering.

### Experimental implants

T-shaped implants (external dimensions: 5.0 mm H ×3.0 mm W ×2.0 mm D) [30], [31] were fabricated from Ti4V6Al titanium alloy and had a hollow inner chamber (3.0 mm H ×3.0 mm W ×1.0 mm D) (**Figure 1A**). The implant surface was treated by dual acid-etching and received the discrete crystalline deposition of HA nanoparticles (Biomet3i, Palm Beach Gardens, FL) [32]. Each implant was gas sterilized and packaged separately.

### Osteotomy and implant placement

Fourteen-week-old male Sprague-Dawley rats were fed with either standard (#7013, Harlan Teklad, Madison, WI) or vitamin D-deficient diet (0.47% Ca, 0.3% P) (#TD.89123, Harlan Teklad) [29]. Both the control and the vitamin D-deficient groups were housed in 12hr-dark/light cycles with fluorescent lighting lacking ultraviolet B (UVB) irradiance. After 4 weeks, rats were further assigned to the following four experimental groups: OSV+ (control rats received osteotomy without the implant placement), OSV- (vitamin-D deficient rats received osteotomy without the implant placement), ITV+ (control rats received osteotomy and the implant placement), and ITV- (vitamin D deficient rats received osteotomy and the implant placement). Under 2% isoflurane inhalation anesthesia, the implant site was prepared at 7 mm to 10 mm from the distal edge of the femur by a slow speed rotary instrument under constant irrigation with sterile 0.9% NaCl solution (**Figure 1A**). For the ITV+ and ITV- groups, a T-shaped implant was placed into the osteotomy site until the implant roof structure reached the femur exterior surface. For the OSV+ and OSV- groups, an osteotomy with a size and depth same as the implant dimensions was created without implant placement. Animals were given 0.012 mg buprenorphine hydrochloride (Buprenex Injectable, Reckitt Benckiser Healthcare Ltd., Hull, England) after surgery, and drinking water was supplemented with 400 mg Sulfamethoxalole and 80 mg Trimethoprim (HI-TECH Pharmacal Co. Inc., Amityville, NY)/500 ml water for 7 days.

### Non-decalcified histology

Two weeks after the implant placement, the implant-femur specimens of ITV+ (*n* = 4) and ITV- (*n* = 4) groups were harvested and fixed in 10% buffered formalin. The tissue samples were processed for histology without decalcification. Sections were cut perpendicular to the T-shaped implant using motorized microtome with diamond saw and stained with Goldner's Masson Trichrome protocol. Bone volume and bone-implant contact area were measured within the inner chamber space using the histomorphometric software (ImageJ-67, National Institutes of Health, http://rsb.info.nih.gov/ij/).

### Serum chemistry

At the time of sacrifice (6 weeks of the experimental period in the OSV+, OSV-, ITV+ and ITV- groups), approximately 5–6 ml of blood was extracted from each rat. Serum samples were prepared and submitted to following measurements: 25-hydroxycholecalciferol (25D), calcium, phosphorus, magnesium, glucose, triglycerides and parathyroid hormone (PTH).

### Whole-genome microarray assay

Two weeks after implant placement, tissue samples inside the implant hollow chamber were harvested under RNase-free condition (ITV+: *n* = 4; ITV-: *n* = 4). Similarly, tissue samples from osteotomy sites were harvested from the separate groups that underwent the osteotomy but not implant placement (OSV+: *n* = 4; OSV-: *n* = 4). The tissue sampling was consistently performed between 10 am to 12 noon of the day. Subsequently, the total RNA was extracted (miRNeasy Mini kit, Qiagen, Valencia, CA, US) resulting in 16 independent total RNA samples. The RNA concentration was quantified (NanoDropTM 1000 Spectrophotometer, Thermo Scientific, Wilmington, DE) and the RNA quality was confirmed (Bioanalyzer 2000 system, Agilent Technologies Inc., Santa Clara, CA).

Total RNA was then subjected to a two-color channel microarray (Agilent Rat Whole Genome, Agilent Technologies, Foster City, CA) in order to screen the transcriptome profiles of more than 41,000 genes. The “reference pool” was prepared by combining 250 ng of the 4 RNA samples from the OSV+ group (total 1 µg) and labeled with the Cy5 fluorescent dye. All the other experimental replicates were individually labeled with the Cy3 fluorescent dye (1 µg of total RNA) and further hybridized with the reference pool sample. In the initial analysis, microarray hybridization and detection underwent rigorous reproducibility tests (data not shown). Microarray signal expression data were obtained using the Agilent Feature Extraction Software (version 7.5.1). We used GeneSifter software (www.genesifter.net) to pre-process and analyze microarray data. Data was expressed as Ratio Cy3/Cy5 instead of Cy3 and Cy5 intensities. LOWESS normalization method [55] was used to improve the reliability of measuring relative differences across samples, improving the precision and sensitivity.

### Microarray Data Analysis Strategy

This study used stringent criteria to identify a network of genes that are associated with the success and failure of implant osseointegration by comparing bone wound healing and peri-implant healing in two conditions: vitamin D sufficiency and deficiency. The whole genome microarray data were further evaluated through the following data analysis strategy: first, the microarray data were analyzed by two-way analysis of valiance (ANOVA) with false discovery rate (FDR) correction to identify significantly modulated gene transcripts for each factor. The identified genes from two-way ANOVA were then categorized into biologically relevant groups using the Kyoto Encyclopedia of Genes and Genomes (KEGG) pathway database. The two-way ANOVA-identified genes were also separately evaluated by hierarchical cluster analysis. Finally, a hypothetical mechanistic model was established, which was tested *in vitro*.

### Two-way ANOVA

Because our experiment design considered two factors: the healing type (OS and IT) and the vitamin D condition (V+ and V-), the two-way ANOVA evaluation was first applied for data analysis. For interpretation of microarray data, it is important to reduce false discoveries (type I errors) and increase the power to detect differences in gene expression among the groups [56]. To this end, a list of differently expressed genes was obtained from 4 independent biological replicates in each group after the two-way ANOVA P-values were adjusted for FDR with Benjamini and Hochberg correction [57] with 95% confidence interval (*P*_cutoff_ = 0.05). We considered the threshold of 2.0-fold change for differently expressed transcripts and *P*<0.05 was considered significant.

### Kyoto Encyclopedia of Genes and Genomes (KEGG) Pathway analysis

Differently expressed transcriptomes were next analyzed by KEGG Pathway analysis (http://www.genome.jp/kegg) to assign genes into known biological pathways and reaction networks. Pathways showing *z*-score above two (*z*-score ≥2.0) were identified.

### Hierarchical clustering analysis

All transcriptomes above 2.0-fold change by two-way ANOVA were grouped into clusters. The hierarchical clustering was based on Euclidean-Distance. Bootstrapping supported 1,000 dataset resampling to generate consensus clusters. The hierarchical cluster analysis was performed using MeV v4.3.01 (MultiExperiment Viewer, TM4 Microarray Software Suite) (http://www.tm4.org/mev.html).

### RTPCR confirmation

RNA samples used for microarray study were tested with real time reverse transcription polymerase chain reaction (RTPCR) using commercially available Taqman-based primer/probe cocktails for selected genes representing bone extracellular matrix (ECM): Col1a2 (Rn00670286_m1), osteonectin (Rn01470624_m1), osteopontin (Rn00681031_m1); cartilage ECM: Col2a1 (Rn01637087_m1); and circadian rhythm: Per2 (Rn01427704_m1). For Col10a1, the primer/probe cocktail was customly designed based on ref. XM_001053056.1 (Applied Biosystems, Carlsbad, CA).

### Bone marrow mesenchymal stem cell culture on titanium disks and vitamin D supplementation

Mouse bone marrow derived mesenchymal stem cells (D1 ORL UVA [D1], ATCC® Number: CRL-s12424™) were plated at a density of 16,000 cells/well either on 48-well culture dishes (Becton Dickinson Labware, Franklin Lakes, NJ) or on sterile 10-mm Ti discs with surface of dual acid-etching and discrete crystalline deposition of HA nanoparticles (Biomet3I) [32]. D1 cells were maintained with minimum Dulbecco's Modified Eagle's medium (1X DMEM, Cellgro, Mediatech Inc., Manassas, VA) supplemented with 10% fetal bovine serum (FBS, Benchmark, Gemini Bio-Products, West Sacramento, CA), and 1% penicilin-streptomicin (PS, MD Biomedicals, Thermo Fisher Scientific) under a humidified atmosphere of 5% CO_2_ in air, 37°C. One day after the cell seeding, the media was changed to osteogenic differentiation medium (1X DMEM, 10% FBS, 1% PS, 10 mM beta glycerol phosphate and 50 µM ascorbic acid) with or without supplementation of 10 nM 1-alpha, 25-Dihydroxy-Vitamin D3 (1,25D, EMD Biosciences Inc., San Diego, CA). After 1, 3, 7 and 14 days of culturing in differentiation medium with or without vitamin D supplementation, RNA was isolated from the cells (RNeasy Mini kit, Qiagen). RT-PCR was performed using Taqman protocol with commercially available primer/probe cocktails (Applied Biosystems, Carlsbad, CA), for Col1a2 (Mm00483937_m1), osteonectin (Mm00486332_m1), Runx2 (Mm0302249_g1), Col2a1 (Mm01309565_m1), Col10a1 (Mm00487041_m1), Sox9 (Mm00448840_m1) and NPAS2 (Mm00500848_m1). Glyceraldehyde 3-dehydrogenase (GAPDH, cat#4352338E or cat#Mm99999915_g1, Applied Biosystems) was used as internal control.

In the separate study, the effect of NPAS2 knockdown on the expression of cartilage ECM genes was examined. D1 cells were cultured with or without the implant disc (10,000 cells/well). On the second day, cells were incubated with commercially available siRNA targeting NPAS2 or negative control siRNA (ID# s70729 and #4390843, respectively, Applied Biosystems) in the final concentration of 23 nM with Lipofectamin 2000 (Invitrogen, Carlsbad, CA). After 14 hours of siRNA incubation, cells were washed and cultured for additional 2 days in the osteogenic differentiation medium with 10 nM 1,25D supplementation. RNA was isolated from the cells and RT-PCR was performed for NPAS2, Col2a1, and Col10a1.

### Immunological identification of type X collagen within the implant osseointegration

For the *in vitro* study, D1 cells were cultured in glass chamber slides (Lab Tek, Fisher Scientific, Pittsburgh, PA) or on titanium disks under the same conditions described above. After 3, 7 and 14 days of culturing, cells were fixed with freshly prepared 3.7% formaldehyde (methanol free) followed by the treatment with 0.2% Triton X-100 in PBS glycine calcium and magnesium free (three washes for 5 minutes) and Image-IT FX signal enhancer for 30 minutes (Invitrogen, Carlsbad, CA). The slides were incubated over night with anti-type X collagen antibody (dilution 1∶50, ab58632, Abcam Inc., Cambridge, MA) followed by Alexa Fluor 488-conjugated secondary antibody for 45 minutes (dilution 1∶500, Invitrogen, Carlsbad, CA). To highlight cytoplasmic area, specimens were incubated over night with anti-b actin antibody (dilution 1∶200, ab6276, Abcam Inc) followed by Alexa Fluor 568-conjugated secondary antibodies for 45 minutes (dilution 1∶500, Invitrogen) or some specimens were incubated with Alexa Fluor 568-conjugated phalloidin (dilution 1∶200). Primary and secondary antibodies were diluted in 10% FBS, 0.5% BSA, 0.01% Triton X-100 in calcium and magnesium free PBS. Samples were washed three times in PBS calcium and magnesium free for five minutes each after each incubation time. The slides were further treated with DAPI (in mounting medium ProLong® Gold antifade reagent, Cell Signaling, Invitrogen) and examined with a confocal laser scanning microscope (Leica SP2 1P-FCS, Leica Camera AG, Solms, Germany).

Confocal laser scanning micrographs were further subjected to cell size measurement. The area of cytoplasma was depicted by cytoskeleton structure resulted from b-actin immunostaining. The peripheral size of the cellular cytoplasma was measured from at least 10 cells in each group using a digital image software (ImageJ 1.43, NIH, Bethesda, MD, USA). The data were compared by Student's t test with p<0.05 as significant.

For the *in vivo* study, T-shape implants (n = 4) were surgically placed in rat femur as described above. After 2 weeks of healing, implants were harvested and stored in RNA stabilizing reagent (RNAlater, Ambion, Austin, TX) under −80°C. It has been shown that the histology of tissues can be preserved by the RNA stabilizing agent [58]. After gradual thawing, large bone tissues were gently removed from the external surface of the implant without scratching the surface. The T-shape implants with remaining tissues were fixed for 15 minutes with freshly prepared 3.7% formaldehyde (methanol free). The method involving type X collagen immunostaining and confocal laser scanning microscopy was described above.

## References

[1] Albrektsson T, Albrektsson B (1987). Osseointegration of bone implants. A review of an alternative mode of fixation.. Acta Orthop Scand.

[2] Marco F, Milena F, Gianluca G, Vittoria O (2005). Peri-implant osteogenesis in health and osteoporosis.. Micron.

[3] Cooper LF, Masuda T, Yliheikkila PK, Felton DA (1998). Generalizations regarding the process and phenomenon of osseointegration. Part II. In vitro studies.. Int J Oral Maxillofac Implants.

[4] Davies JE (2003). Understanding peri-implant endosseous healing.. J Dent Educ.

[5] Schwartz Z, Nasazky E, Boyan BD (2005). Surface microtopography regulates osteointegration: the role of implant surface microtopography in osteointegration.. Alpha Omegan.

[6] Carinci F, Pezzetti F, Volinia S, Francioso F, Arcelli D (2004). Analysis of MG63 osteoblastic-cell response to a new nanoporous implant surface by means of a microarray technology.. Clin Oral Implants Res.

[7] Carinci F, Pezzetti F, Volinia S, Francioso F, Arcelli D (2003). Analysis of osteoblast-like MG63 cells' response to a rough implant surface by means of DNA microarray.. J Oral Implantol.

[8] Kim CS, Sohn SH, Jeon SK, Kim KN, Ryu JJ (2006). Effect of various implant coatings on biological responses in MG63 using cDNA microarray.. J Oral Rehabil.

[9] Kojima N, Ozawa S, Miyata Y, Hasegawa H, Tanaka Y (2008). High-throughput gene expression analysis in bone healing around titanium implants by DNA microarray.. Clin Oral Implants Res.

[10] Alvim-Pereira F, Montes CC, Mira MT, Trevilatto PC (2008). Genetic susceptibility to dental implant failure: a critical review.. Int J Oral Maxillofac Implants.

[11] Holahan CM, Koka S, Kennel KA, Weaver AL, Assad DA (2008). Effect of osteoporotic status on the survival of titanium dental implants.. Int J Oral Maxillofac Implants.

[12] Lekholm U (2003). Immediate/early loading of oral implants in compromised patients.. Periodontol 2000.

[13] Econs MJ, Lobaugh B, Drezner MK (1992). Normal calcitonin stimulation of serum calcitriol in patients with X-linked hypophosphatemic rickets.. J Clin Endocrinol Metab.

[14] Kelly J, Lin A, Wang CJ, Park S, Nishimura I (2009). Vitamin D and bone physiology: demonstration of vitamin D deficiency in an implant osseointegration rat model.. J Prosthodont.

[15] Lin R, White JH (2004). The pleiotropic actions of vitamin D.. Bioessays.

[16] Slominski A, Wortsman J (2000). Neuroendocrinology of the skin.. Endocr Rev.

[17] Adams JS (2006). Vitamin D as a defensin.. J Musculoskelet Neuronal Interact.

[18] Adams JS, Hewison M (2008). Unexpected actions of vitamin D: new perspectives on the regulation of innate and adaptive immunity.. Nat Clin Pract Endocrinol Metab.

[19] Misra M, Pacaud D, Petryk A, Collett-Solberg PF, Kappy M (2008). Vitamin D deficiency in children and its management: review of current knowledge and recommendations.. Pediatrics.

[20] Bar-Shavit Z, Kahn AJ, Teitelbaum SL (1983). Defective binding of macrophages to bone in rodent osteomalacia and vitamin D deficiency. In vitro evidence for a cellular defect and altered saccharides in the bone matrix.. J Clin Invest.

[21] Heaney RP (2003). Long-latency deficiency disease: insights from calcium and vitamin D.. Am J Clin Nutr.

[22] Reginster JY (2005). The high prevalence of inadequate serum vitamin D levels and implications for bone health.. Curr Med Res Opin.

[23] de Abreu DA, Eyles D, Feron F (2009). Vitamin D, a neuro-immunomodulator: implications for neurodegenerative and autoimmune diseases.. Psychoneuroendocrinology.

[24] Liu PT, Stenger S, Li H, Wenzel L, Tan BH (2006). Toll-like receptor triggering of a vitamin D-mediated human antimicrobial response.. Science.

[25] Holick MF (2006). Resurrection of vitamin D deficiency and rickets.. J Clin Invest.

[26] Mithal A, Wahl DA, Bonjour JP, Burckhardt P, Dawson-Hughes B (2009). Global vitamin D status and determinants of hypovitaminosis D.. Osteoporos Int.

[27] Hirani V, Tull K, Ali A, Mindell J (2010). Urgent action needed to improve vitamin D status among older people in England!. Age Ageing.

[28] Saintonge S, Bang H, Gerber LM (2009). Implications of a new definition of vitamin D deficiency in a multiracial us adolescent population: the National Health and Nutrition Examination Survey III.. Pediatrics.

[29] Hokugo A, Christensen R, Chung E, Sung E, Felsenfeld AL (2010). Increased prevalence of bisphosphonate-related osteonecrosis of the jaw with vitamin D deficiency in rats.. J Bone Miner Res Epub ahead of print.

[30] Mendes VC, Moineddin R, Davies JE (2009). Discrete calcium phosphate nanocrystalline deposition enhances osteoconduction on titanium-based implant surfaces.. J Biomed Mater Res A.

[31] Ozawa S, Ogawa T, Iida K, Sukotjo C, Hasegawa H (2002). Ovariectomy hinders the early stage of bone-implant integration: histomorphometric, biomechanical, and molecular analyses.. Bone.

[32] Nishimura I, Huang Y, Butz F, Ogawa T, Lin L (2007). Discrete deposition substrate microtopography accelerated osseointegration.. Nanotechnology.

[33] Ogata H, Goto S, Sato K, Fujibuchi W, Bono H (1999). KEGG: Kyoto Encyclopedia of Genes and Genomes.. Nucleic Acids Res.

[34] Takahashi JS, Hong HK, Ko CH, McDearmon EL (2008). The genetics of mammalian circadian order and disorder: implications for physiology and disease.. Nat Rev Genet.

[35] Panda S, Antoch MP, Miller BH, Su AI, Schook AB (2002). Coordinated transcription of key pathways in the mouse by the circadian clock.. Cell.

[36] Schibler U (2009). The 2008 Pittendrigh/Aschoff lecture: peripheral phase coordination in the mammalian circadian timing system.. J Biol Rhythms.

[37] Fu L, Patel MS, Bradley A, Wagner EF, Karsenty G (2005). The molecular clock mediates leptin-regulated bone formation.. Cell.

[38] Miller BH, McDearmon EL, Panda S, Hayes KR, Zhang J (2007). Circadian and CLOCK-controlled regulation of the mouse transcriptome and cell proliferation.. Proc Natl Acad Sci U S A.

[39] Borgs L, Beukelaers P, Vandenbosch R, Belachew S, Nguyen L (2009). Cell “circadian” cycle: new role for mammalian core clock genes.. Cell Cycle.

[40] Lin A, Wang CJ, Kelly J, Gubbi P, Nishimura I (2009). The role of titanium implant surface modification with hydroxyapatite nanoparticles in progressive early bone-implant fixation in vivo.. Int J Oral Maxillofac Implants.

[41] Mendes VC, Moineddin R, Davies JE (2007). The effect of discrete calcium phosphate nanocrystals on bone-bonding to titanium surfaces.. Biomaterials.

[42] Iwasa F, Hori N, Ueno T, Minamikawa H, Yamada M (2009). Enhancement of osteoblast adhesion to UV-photofunctionalized titanium via an electrostatic mechanism.. Biomaterials.

[43] Le Guehennec L, Lopez-Heredia MA, Enkel B, Weiss P, Amouriq Y (2008). Osteoblastic cell behaviour on different titanium implant surfaces.. Acta Biomater.

[44] Gu YZ, Hogenesch JB, Bradfield CA (2000). The PAS superfamily: sensors of environmental and developmental signals.. Annu Rev Pharmacol Toxicol.

[45] McIntosh BE, Hogenesch JB, Bradfield CA (2010). Mammalian Per-Arnt-Sim proteins in environmental adaptation.. Annu Rev Physiol.

[46] Yang X, Downes M, Yu RT, Bookout AL, He W (2006). Nuclear receptor expression links the circadian clock to metabolism.. Cell.

[47] Eisen MB, Spellman PT, Brown PO, Botstein D (1998). Cluster analysis and display of genome-wide expression patterns.. Proc Natl Acad Sci U S A.

[48] Markway BD, Tan GK, Brooke G, Hudson JE, Cooper-White JJ (2009). Enhanced Chondrogenic Differentiation of Human Bone Marrow-Derived Mesenchymal Stem Cells in Low Oxygen Environment Micropellet Cultures.. Cell Transplant.

[49] Khan WS, Adesida AB, Tew SR, Lowe ET, Hardingham TE (2010). Bone marrow-derived mesenchymal stem cells express the pericyte marker 3G5 in culture and show enhanced chondrogenesis in hypoxic conditions.. J Orthop Res.

[50] Schipani E (2005). Hypoxia and HIF-1 alpha in chondrogenesis.. Semin Cell Dev Biol.

[51] Zuscik MJ, Hilton MJ, Zhang X, Chen D, O'Keefe RJ (2008). Regulation of chondrogenesis and chondrocyte differentiation by stress.. J Clin Invest.

[52] Seki K, Fujimori T, Savagner P, Hata A, Aikawa T (2003). Mouse Snail family transcription repressors regulate chondrocyte, extracellular matrix, type II collagen, and aggrecan.. J Biol Chem.

[53] Hinoi E, Takarada T, Fujimori S, Wang L, Iemata M (2007). Nuclear factor E2 p45-related factor 2 negatively regulates chondrogenesis.. Bone.

[54] Kwan AP, Cummings CE, Chapman JA, Grant ME (1991). Macromolecular organization of chicken type X collagen in vitro.. J Cell Biol.

[55] Zahurak M, Parmigiani G, Yu W, Scharpf RB, Berman D (2007). Pre-processing Agilent microarray data.. BMC Bioinformatics.

[56] Devlin B, Roeder K, Wasserman L (2003). False discovery or missed discovery?. Heredity.

[57] Hochberg Y, Benjamini Y (1990). More powerful procedures for multiple significance testing.. Stat Med.

[58] Florell SR, Coffin CM, Holden JA, Zimmermann JW, Gerwels JW (2001). Preservation of RNA for functional genomic studies: a multidisciplinary tumor bank protocol.. Mod Pathol.

